# Prevalence of peripheral neuropathy, amputation, and quality of life in patients with diabetes mellitus

**DOI:** 10.1038/s41598-024-65495-2

**Published:** 2024-06-23

**Authors:** Wajida Perveen, Hafsa Ahsan, Rameen Shahzad, Samra Fayyaz, Ayesha Zaif, Mahnoor Asif Paracha, Shibili Nuhmani, Masood Khan, Ahmad H. Alghadir

**Affiliations:** 1 School of Allied Health Sciences, CMH Lahore Medical College & Institute of Dentistry, (NUMS Rawalpindi), Lahore, Pakistan; 2 Department of Physical Therapy, College of Applied Medical Sciences, Imam Abdulrahman Bin Faisal University, Dammam, Saudi Arabia; 3 Rehabilitation Research Chair, Department of Rehabilitation Sciences, College of Applied Medical Sciences, King Saud University, P.O. Box. 10219, Riyadh-11433 Riyadh, Saudi Arabia

**Keywords:** Peripheral neuropathy, Amputation, Quality of life, Diabetes Mellitus, Michigan Neuropathy Screening Instrument (MNSI), Asian Diabetic Quality of Life Questionnaire (AsianDQOL), Diabetes, Peripheral neuropathies

## Abstract

Peripheral neuropathy and amputation are common complications of diabetes mellitus (DM) that significantly impact the quality of life of the affected individuals. This study aims to investigate the prevalence of peripheral neuropathy, the level of amputation, and the quality of life in patients with DM. This cross-sectional study was conducted after approval of the synopsis involving 225 diagnosed patients with DM on pre-defined eligibility criteria, selected from public sector OPDs, specialized diabetes centres, and centres manufacturing orthotics and prosthetics. Data were collected through interviews, observations, and the administration of the Michigan Neuropathy Screening Instrument and the Asian Diabetes Quality of Life Questionnaire. The level of amputation was recorded for each participant. Data was entered into SPSS, and results were synthesized. Pearson correlation is applied to find an association between gender and the quality of life of the participants, while P ≤ 0.05 will be considered significant. The prevalence of peripheral neuropathy in a sample of 225, based on a self-administered questionnaire, was (44.4%), and in terms of foot examination was (51.1%). As people progressed in age, the prevalence increased to 20.0% in patients above 60 years and 8.9% in ≤ 35 years of age. The majority of participants (56.0%) have had DM for less than five years. Females were 57.8% of the study population, while 97.8% of participants had type II DM. Below-knee amputation of the right limb was observed in 22(9.8%) of the participants. The QoL was poor in the majority of the participants (96.9%) patients with DM (P = 0.638 and T = -0.471). This cross-sectional study highlights a high prevalence of peripheral neuropathy and amputation and poor QoL in patients with diabetic mellitus.

## Introduction

Diabetes Mellitus (DM) is one of the non-communicable illnesses whose incidence is increasing globally. At least half of the patients with DM get Diabetic Neuropathy over time. In recent decades, DM has been more common in industrialized and developing countries. The prevalence of DM has sharply increased worldwide over the last few decades. Diabetes affects an estimated 537 million adults worldwide between the ages of 20 to 79. By 2030, 643 million people will have diabetes globally, increasing to 783 million by 2045^1^.

Diabetes-related lower extremity problems affect roughly 131 million people worldwide. The prevalence of Diabetes in Pakistan was 11.77% in 2016, 16.98% in 2018 and 17.1% in 2019; this growing prevalence makes Pakistan among the top 10 countries in the world^2^. At least half of the patients with DM get diabetic peripheral neuropathy (DPN) over time. Glucose control effectively slows the course of DPN in type I patients with DM , but the effect is smaller in type II patients with DM , who constitute the majority^3,4^. Chronic hyperglycemia, which is associated with poorly controlled DM, damages numerous organs and causes chronic diabetic complications, which can lead to physical or mental incapacities, decreased quality of life, and eventually death of the patient. Diabetic foot is one of the most prevalent complications observed in DM ^5^.

Patients with DM exhibit symptoms ranging from unexpected weight loss to increased urine, thirst, and hunger. Among these symptoms, neuropathy is a prevalent and major one, affecting 90% of patients^6^. Navarro-Flores et al. (2020) stated that Diabetic foot syndrome (DFS) is a common chronic consequence of DM and can negatively affect the overall well-being of these patients, leading to impairment in their quality of life^7^. J. Aaron Barnes et al. (2020) narrated that Peripheral artery disease (PAD), which has a high risk of amputation and cardiovascular death, is caused by atherosclerosis of the arteries in the lower extremities. Amputation rates among people with both DM and PAD have remained consistent or even increased in high-risk subgroups despite a decline in the overall number of operations^8^.

Sorber R et al. (2021) described that Diabetic foot ulcers (DFUs) are severe complications that commonly arise in individuals with long-standing DM, often leading to high rates of major amputation and mortality and needing special attention in those with known peripheral neuropathy^9^. In a retrospective population-based cohort analysis, Kamitani F et al. (2021) studied the rates and trends of lower limb amputation (LLA) in adults with and without DM using national claims data from Japan on major and minor LLA rates over five years. The incidence of major and minor LLAs was significantly higher among individuals with DM^10^.

Lower extremity amputations caused by this condition significantly reduce the patient's quality of life and function and significantly raise the healthcare cost. Amputation rates in Pakistan are also high (21%-48%) due to patient non-compliance, poor glycemic control and inadequate early care of foot ulcers^2^. Amputation results in the loss of independence and livelihood and has a catastrophic effect on the individual and their families. These complications have a noteworthy impact on the quality of life and psychological status of patients with DM. As a result, the patient's quality of life has been acknowledged as a measure of treatment effect^11^.

Diabetic peripheral neuropathy (DPN) is characterized by two key clinical outcomes: diabetic foot ulceration and neuropathic pain^12^. The “glove and stocking” sensory loss occurs because of the distal symmetric sensorimotor polyneuropathy^13^. Martin CL et al. in 2014 found that the most common of these neuropathic disorders is chronic diabetic peripheral sensorimotor neuropathy (DPN), which affects up to 50% of persons with DM^14^. Hazari et al. found that individuals with type II DM and DPN have prominent changes in their foot kinematics and kinetics, putting them at a higher risk for foot ulceration with underlying neuropathy and biomechanical manifestations^15^. A higher degree of functional impairment, a reduction in health-related quality of life, and difficulties with everyday tasks are all linked to painful DPN^16,17^.

Therefore, it is crucial to determine the health-related quality of life (HRQOL) of patients with DPN and research the solutions that can enhance their HRQOL^18^. The Baqai Institute of Diabetology and Endocrinology (BIDE), a tertiary care diabetic centre that founded Pakistan's first management program, has improved QOL as one of its primary objectives^19^.

Dinesh Selvarajah et al. (2019) described that Diabetic peripheral neuropathy (DPN) is a significant contributor to lower-limb amputations and debilitating neuropathic pain. The impact of amputations in patients with DM is severe, leading to a profound deterioration in their quality of life. Furthermore, individuals who undergo amputations due to DM face alarmingly low life expectancy, with an average survival of only two years following the procedure^20^. The financial burden imposed on healthcare systems and society as a whole due to amputations is also substantial ^20^.

Existing literature has provided insights into the prevalence of peripheral neuropathy. However, still, regarding the amputation rates in patients with DM, there remains a noticeable research gap in understanding the relationship between these complications and their collective impact on the quality of life. The aim is to fill a gap in the existing knowledge by providing an understanding of the prevalence of peripheral neuropathy and amputation in patients with DM while concurrently assessing their quality of life. Therefore, the present study aimed to find the prevalence of peripheral neuropathy, measure the ratio of amputations, and study the quality of life in patients with DM.

## Methods

The cross-sectional study was conducted in the Outpatient Departments (OPD) of Services Hospital Lahore, Pakistan Society for the Rehabilitation of the Disabled (PSRD), and Sheikh Zayed Hospital Lahore, which attends to patients with DM after ethical approval in three months. A sample size of 225 patients was determined^21^ using the formula;

$$x = \frac{{Z^{2} \left( {1 - \frac{\alpha }{2}} \right)P\left( {1 - P} \right)}}{{d^{2} }}$$

P represents the prevalence of peripheral neuropathy = 17.68%,

Z at 1.96 for a 95% confidence interval, and d denoting the marginal error = 0.05.

Non-probability convenient sampling techniques were utilized for participant selection.

Patients diagnosed with DM aged between 35 and 70, as this demographic exhibited a high prevalence of the condition, were included, as were both male and female patients who had either Type I or Type II DM cases and were willing to participate in the study. Individuals with concurrent chronic illnesses like heart disease, cancer, or renal disorders, with a history of trauma, severe psychiatric conditions, or addiction were excluded. Additionally, patients with comorbidities related to neurological disorders, malignancies, nerve root compression, or non-diabetic peripheral neuropathy-associated pain conditions were not considered. Cases of amputation due to trauma, tumours, congenital limb deficiencies, or infection were also excluded.

**Figure 1:**
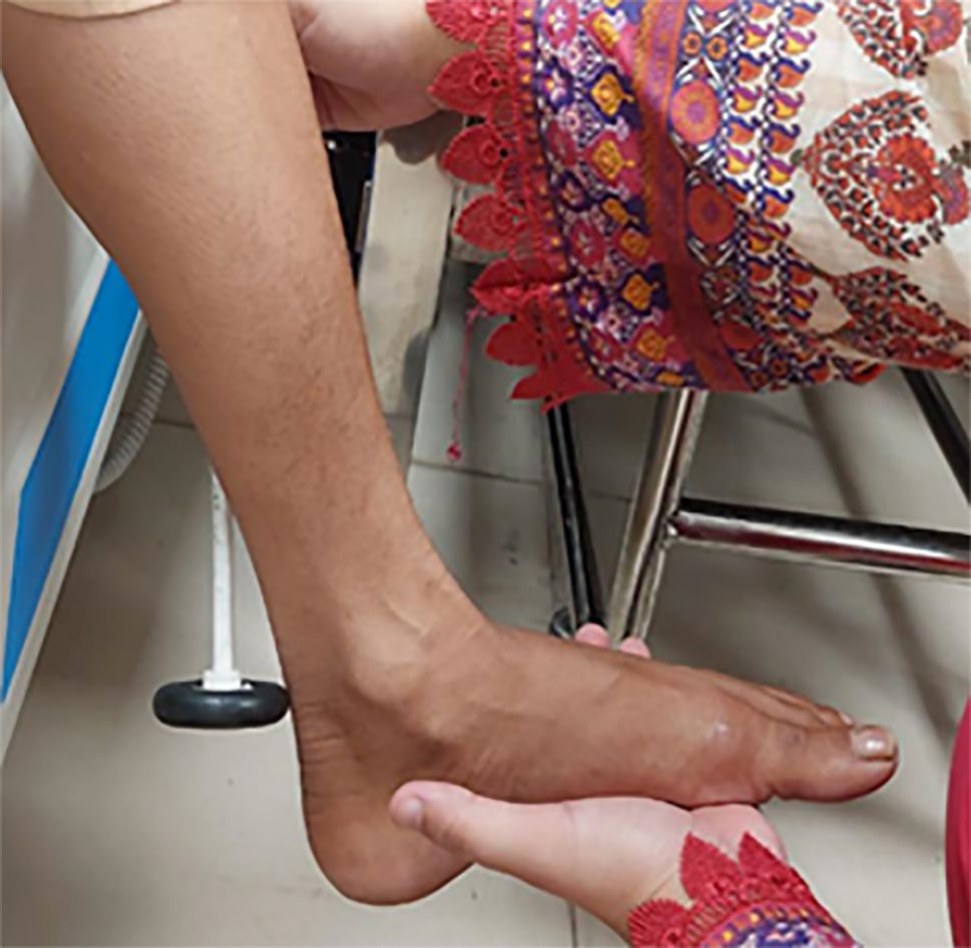
Clinical hammer.

**Figure 2:**
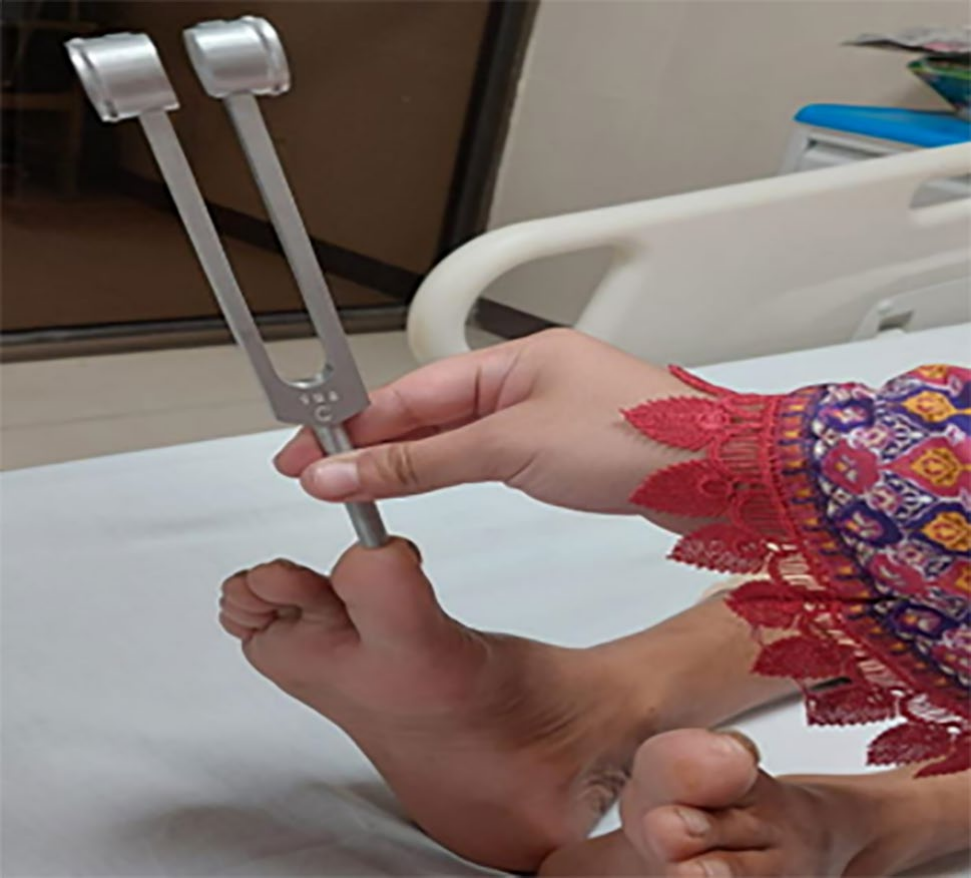
Tuning fork.

**Figure 3:**
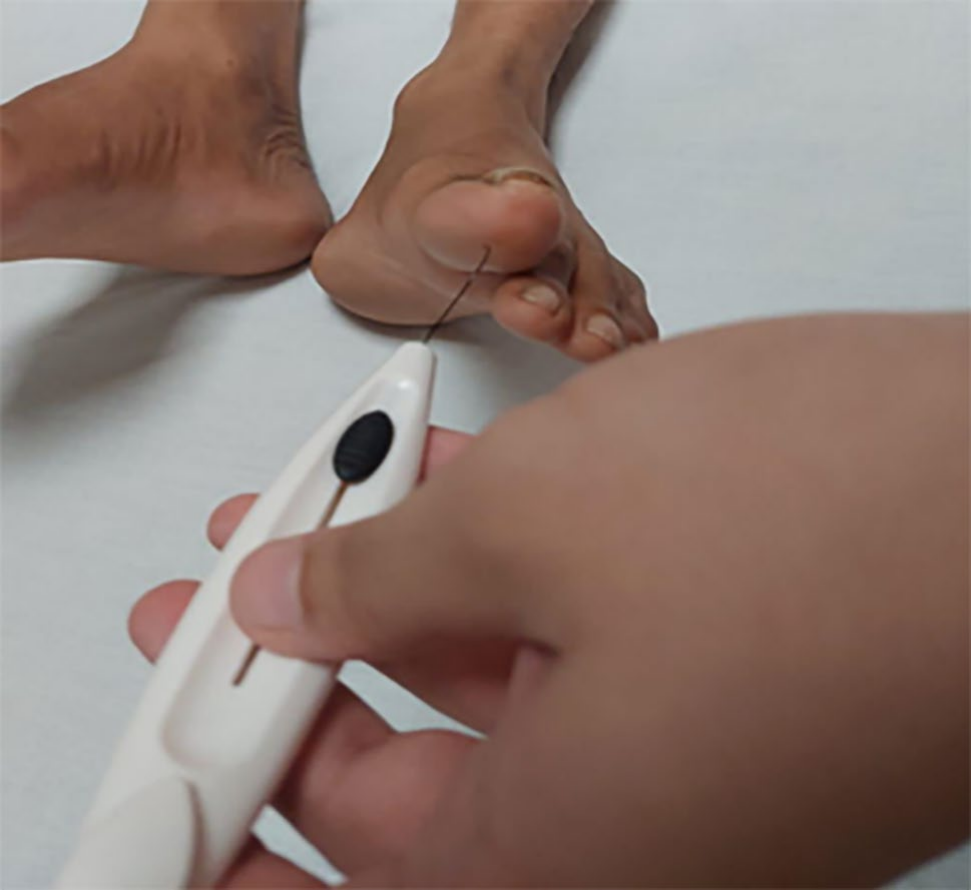
Monofilament wire.

Outcome measurements relied on the Michigan Neuropathy Screening Instrument (MNSI), known for its 80% sensitivity and 95% specificity. The MNSI consisted of two parts: a self-administered questionnaire regarding Diabetic Peripheral Neuropathy (DPN) symptoms and a brief physical examination of the foot. The study employed specific equipment for assessment, including a 128 Hz frequency tuning fork with a sensitivity of 72.5% and specificity of 88.7%, a clinical hammer with a sensitivity of 51.4% and specificity of 97.7%, and a 10-g SWM diabetic monofilament with a sensitivity of 69.7% and specificity of 87.9%^22–24^ but have a limited accuracy in Indian population, further affected by occupation, socioeconomic status and religious practices^25^ Figs. (1,2,3). The questionnaire part has a cut-off value of ≥ 7, and the foot examination part has a cut-off value of ≥ 2.5 for DPN to be positive^26^. Additionally, the Asian Diabetes Quality of Life Questionnaire (Asian DQOL) was employed, utilizing a total score range of 0–60. The analysis categorized scores into poor, moderate, good, or excellent, providing insights into the participants' quality of life^27^.

Quality of life, Amputation and Peripheral neuropathy are dependent, while DM is an independent variable. Data is analyzed using SPSS version 26.0. Quantitative variables are given as mean ± standard deviation. Qualitative variables are expressed as ratios and percentages. Pearson correlation is applied to find an association between gender and the quality of life of the participants, while P ≤ 0.05 will be considered significant.

## Results

The mean age of participants was 54.08, with a standard deviation of 8.98 years ranging from 35 to 70 years. The majority of the patients had type-II DM (97.78%) (type-I DM 2.22%); out of them, 57.78% were female, and 42.22% were male participants. The demographic and clinical characteristics of the participants are given in Table 1.

**Table 1:** Demographic and Clinical Characteristics of Patients with Diabetes Mellitus.

Variables		No of Participants	(%)
Age of participants (Years) Mean ± SD 54.08 ± 8.98	35–40	20	8.9
	41–45	29	12.9
	46–50	36	16.0
	51–55	43	19.1
	56–60	30	13.3
	61–65	45	20.0
	66–70	22	9.8
H/O DM for years	< 5	126	56.0
	5–10	72	32.0
	11–15	15	6.70
	16–20	9	4.0
	> 20	3	1.3
Gender	Male	95	42.2
	Female	130	57.8
Types of Diabetes Mellitus	Type I	5	2.2
	Type II	220	97.8

*SD* standard deviation.

Table 1 shows the mean ± standard deviation age of participants along with the number of participants in different age groups. More than half of the participants, 126 (56%), have had DM for less than five years, and 1.3% of the participants have had DM for more than two decades. Female gender was dominant among the study participants, with one hundred and thirty participants (57.8%).

**Figure 4:**
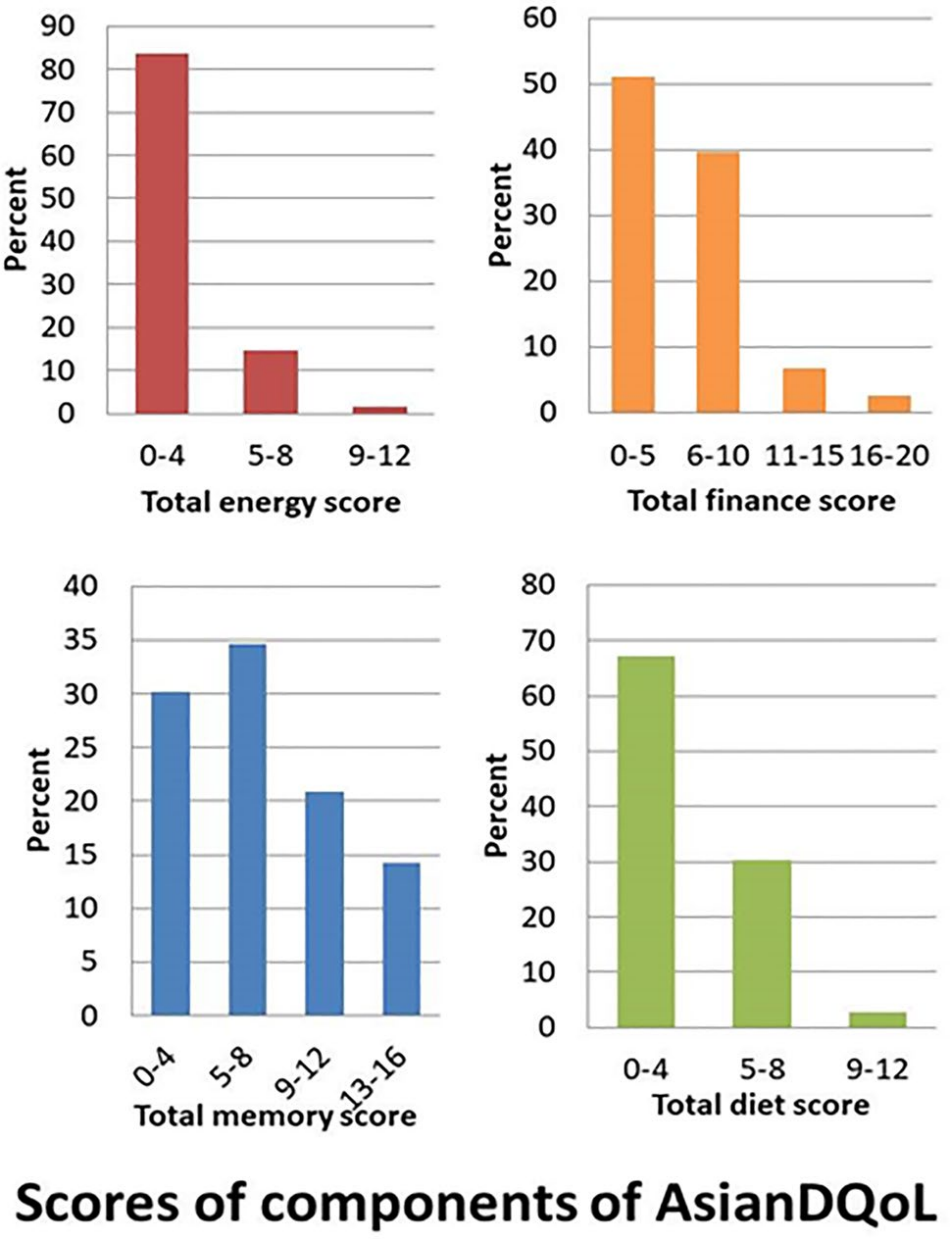
Scores of components of AsianDQoL.

Table 2 and Fig. 4 show scores of components of the AsianDQoL questionnaire. According to scores of individual components of the AsianDQoL questionnaire, the total Energy score ranged from 0–12 with a mean score 2.81 ± 1.82 standard deviation; the total Memory score ranged 0–16 with mean of 7.97 ± 4.53 standard deviation, the total Finance score ranged 0–20 with mean 6.38 ± 4.34 std dev, total Diet score ranged 0–12 with mean 3.77 ± 2.17 standard deviation. The total score of AsianDQoL ranged from 0–60, with a mean of 20.95 and a standard deviation of 10.4.

**Table 2:** Scores of Components of Asian Diabetes Quality of Life (AsianDQOL) questionnaire.

Components	Range	No of participants	(%)	Mean ± SD
Total energy score 0–12	0–4	188	83.6	2.81 ± 1.82
	5–8	33	14.7	
	9–12	4	1.8	
Total memory score 0–16	0–4	68	30.2	7.97 ± 4.53
	5–8	78	34.7	
	9–12	47	20.9	
	13–16	32	14.2	
Total finance score 0–20	0–5	115	51.1	6.38 ± 4.34
	6–10	89	39.6	
	11–15	15	6.7	
	16–20	6	2.7	
Total diet score 0–12	0–4	151	67.1	3.77 ± 2.17
	5–8	68	30.2	
	9–12	6	2.7	
The total score of AsianDQOL 0–60	Below 45	218	96.9	20.95 ± 10.40
	45–50	5	2.2	
	50–55	2	0.9	

*SD* standard deviation.

Table 2 also shows the number of participants in subcategories of scores in each category of subsets of the AsialDQOL. In the total energy score, 188 participants of categories 0–4 signify 83.6%, 33 participants of the 5–8 category signify 14.7%, and 4 participants of the 9–12 category signify a 1.8% decrease in quality of life. In the total memory score variable, 68 participants of category 0–4 show 30.2%, 78 participants of the 5–8 category show 34.7%, 47 participants of the 9–12 category show 20%, 32 participants of the 13–16 category show 14.2% of the population quality of life has been affected. In the total finance score variable, category 0–5 with 115 participants indicates 51.1%, category 6–10 with 89 participants shows 39.6%, 11–15 category with 15 participants shows 6.7%, category 16–20 with 6 participants shows 2.7% population quality of life has been affected. In the Total Diet Score variable, a sample of 151 in categories 0–4 indicates 67.1%, a sample of 68 in categories 5–8 indicates 30.3, and a sample of 6 in categories 9–12 indicates 2.7% of the population's quality of life has been affected. In the total AsianDQol score variable, the sample below 45 is 218(96.6%), showing poor quality of life; five participants (2.2%) have a score of 45–50 moderate and only two (0.9%) fall in the category of score 50–55 reflecting a better QOL.

Table 3 shows a comparison of the quality of life in both genders. The male participants' total mean energy score is 2.78 with ± standard deviation of 1.82 (T = -0.168 and P = 0.867). The total memory score means the value is 8.21 with ± standard deviation of 4.59 (T = -0.657 P = 0.512). The total finance score means the value is 5.81 with ± standard deviation of 3.89 (T = -1.695 and P = 0.091). The total diet score mean value is 3.75 with ± standard deviation of 2.02 with T = -0.117 and P = 0.907). The variable total AsianDQoL score mean value is 20.57 with ± standard deviation of 9.75 (T = -0.471 and P = 0.638). Among females, the total energy score mean value is 2.83 with ± standard deviation of 1.82 (T = -0.168 and P = 0.867). The total memory score mean value is 7.80 with ± standard deviation of 4.51 (T = 0.657 and P = 0.512). Total finance score means the value is 6.80 with ± standard deviation of 4.61 (T = -1.695 and P = 0.091). The total diet score mean value is 3.79 with ± standard deviation of 2.28 with (T = 0.117 and P = 0.907). The total AsianDQoL score mean value is 21.23 with ± standard deviation of 10.87 with T = 0.471 and P = 0.638).

**Table 3:** Comparison of Quality of Life (AsianDQoL) in Male and Female Participants.

Variables	Male n (95)	Female n (130)	T Value	P Value
	Mean ± S.D			
Energy score	2.78 ± 1.82	2.83 ± 1.82	− 0.168	0.867
Memory score	8.21 ± 4.59	7.80 ± 4.51	0.657	0.512
Finance score	5.81 ± 3.89	6.80 ± 4.61	− 1.695	0.091
Diet score	3.75 ± 2.02	3.79 ± 2.28	− 0.117	0.907
AsianDQoL	20.57 ± 9.75	21.23 ± 10.87	− 0.471	0.638

*SD* standard deviation, *AsianDQoL* asian diabetes quality of life.

Table 4 shows that according to MNSI questionnaire findings, 125 (55.6%) participants had a score of less than 7, reflecting absence of peripheral neuropathy, while 100 (44.4%) participants had a score of more than 7, indicating the presence of peripheral neuropathy in participants with DM. While on the basis of clinical examination, 115 (51.1%) participants having scores equal to or greater than 2.5 indicated the presence of peripheral neuropathy.

**Table 4:** Prevalence of Peripheral Neuropathy.

	Category	No of participants	(%)
Based on MNSI questionnaire part	(< 7) No	125	55.6
	(≥ 7) Yes	100	44.4
Based on foot examination	(≤ 2.5) No	110	48.9
	(≥ 2.5) Yes	115	51.1

*MNSI* michigan neuropathy screening instrument.

Table 5 shows that the majority of the participants, 194 (86.2%), had no amputation. Only one participant (0.4%) undergone a hemipelvectomy, one participant had shorts above knee amputation, 22 (9.8%) participants had below knee amputation, one had Syme’s amputation and 6 (2.75%) participants underwent toe disarticulation due to DM of their right lower limb. While in the left lower limb, there was no amputation in 207 (92%) participants, shorts above knee amputation in 5 (2.2%) participants, standard below knee amputation in 8 (3.6%) participants, Syme's amputation in 1 (0.4%) participant, toe disarticulation in 4 (1.8%) participants. Overall, the standard below-knee amputation was observed at most in 22 (9.8%) participants on the right side.

**Table 5:** Level of amputation in the right and left leg.

Amputation levels	Level	No. of participants	(%)
Right leg	None	194	86.2
	Hemipelvectomy	1	0.4
	Short above knee	1	0.4
	Standard below knee	22	9.8
	Symes	1	0.4
	Toe disarticulation or amputation	6	2.7
Left leg	None	207	92.0
	Short above knee	5	2.2
	Standard below knee	8	3.6
	Symes	1	0.4
	Toe disarticulation or amputation	4	1.8

### Ethics approval and consent to participate

The ethical approval was obtained from the ethical review committee, CMH Lahore Medical College & IOD, wide reference no. 627/ERC/CMH/LMC. Before initiating any intervention, the risks and benefits of participation were discussed with all participants, who agreed voluntarily and signed the informed consent form. The World Medical Association's (WMA) 2013 Declaration of Helsinki's ethical guidelines for medical research involving human participants were closely followed in this study.

## Discussion

A cross-sectional observational study was conducted on 225 patients (42.2% males and 57.8% females) with type I (2.2%) and type II DM (97.8%), having a diabetic history of fewer than five years in 56% of participants of age ranging from 35-70 years, The study aimed to scrutinize the prevalence of peripheral neuropathy and amputation in patients with DM and evaluate their quality of life by utilizing the Michigan Neuropathy Screening Instrument and the Asian Diabetic Quality of Life Questionnaire. The levels of amputation from toe amputations to hemipelvectomy were observed.

Martin CL et al. in 2014 found that the most common of these neuropathic disorders is chronic diabetic peripheral sensorimotor neuropathy (DPN), which affects up to 50% of persons with DM^14^. The study's findings revealed a significant prevalence of peripheral neuropathy among the participants, emphasizing the considerable burden of this complication in patients with DM. Our study conducted on 225 patients with DM demonstrates the prevalence of DNP (diabetic peripheral neuropathy) using the Michigan neuropathy screening instrument (MNSI) self-administer questionnaire (SAQ) was 44.4% and 51.1% on the basis of the lower extremity examination part of the MNSI. Whereas, 55.6% and 48.9% were observed for the population without DPN using SAQ and the physical examination part of MNSI, respectively. Peripheral neuropathy is a well-recognized consequence of DM, characterized by nerve damage that can lead to various sensory and motor deficits, including pain, tingling, numbness, loss of sensation, muscle weakness, and impaired balance. The high prevalence rate underscores the need for early detection and effective management strategies to prevent or mitigate its adverse effects on patients’ well-being.

Hazari et al. (2023) reported that the risk of DPN is ethnic origin-dependent in residents of the United Arab Emirates and is high in Arab-origin residents. According to the findings of MNSI, 62% of the participants were screened with DPN^28^. The current study's findings are in line with their results in terms of DPN on the basis of MNSI but are limited in terms of information regarding the ethnicity and geographical background of the participants.

Amputation, another severe complication associated with DM, was also found to be prevalent in the study sample. This outcome raises concerns about the impact of DM on vascular health and underscores the significance of comprehensive diabetic foot care programs. Amputations can have profound physical, psychological, and social implications for individuals, leading to long-term disability and reduced quality of life. The identification of factors such as age, duration of DM, and glycemic control as predictors of peripheral neuropathy and amputation provides valuable insights for risk stratification and targeted interventions. In this study, the prevalence of amputations observed in patients with DM in the right and left lower limb are 0.4% and 0% hemipelvectomy, respectively, with short above knee amputation being 0.4%, 2.2%, respectively, standard below the knee in 9.8%, 3.6% respectively, toe disarticulation or amputation in 2.7%, 1.8 respectively, and Symes being 0.4% in both lower limbs. Baumfeld D et al. in 2018 found that in Pakistan, the rate of amputation (number of amputations due to diabetes per year) has been reported to be 21%-48%, despite the prevalence of diabetic foot ulcerations that is comparable to that of other countries^29^. The total rate of amputation, according to our study among the sample population, is 21.7%, which is similar to other research done before.

Furthermore, the study demonstrated the quality of life of the participants. This finding highlights the multidimensional nature of quality of life and the significant impact that these complications have on various aspects of a patient's well-being. According to the results of our study, DPN and amputation may have a negative association with four components of Asian DQOL in patients with DM. Our study showed that 96.9% of the population had poor QOL (score < 45), 2.2% had moderate QOL (score 45–50), and 0.9% had good QOL (score 50–55). The study concluded results for four components of the Asian DQOL questionnaire with lower scores indicating poor QOL and vice versa. Total energy score 83.6% with (scores of 0–4) 14.7%, and 1.8% (scores 5–8 and 9–12, respectively), total memory scores 0–4, 5–8, 9–12, and 13–16 with 30.0%, 34.7%, 20.9%, and 14.2% respectively, total finance score 0–5, 6–10, 11–15, and 16–20 with 51.1%, 39.6%, 6.7%, and 2.7% respectively, and total diet score of 0–4, 5–8, 9–12 with 67.1%, 30.2%, and 2.7% respectively. The mean energy score is 2.81 ± 1.82 out of 12, which seems to be very low; the mean memory score is 7.97 ± 4.53 out of 16, appearing as less than half of the total score, mean finance score is 6.38 ± 4.34 out of 20 which is also very poor, mean diet score is 3.77 ± 2.17 out of 12 and total score is 20.95 ± 10.40 out of 60. Most of the results depict poor quality of life in the study population.

Physical functioning is often compromised due to the sensory and motor deficits associated with peripheral neuropathy, limiting mobility and impairing daily activities. Psychological well-being is affected by chronic pain, anxiety, depression, and the psychological adjustment to the loss of a limb in the case of amputation. Social interactions may also be impacted as individuals may experience social stigma, reduced participation in social activities, and a sense of isolation. Overall, life satisfaction is significantly diminished as a result of the limitations imposed by these complications. According to our findings, numerous studies from different countries have indicated that type II DM has a detrimental effect on QOL^30–33^.

While this study provides valuable insights into the prevalence of peripheral neuropathy, amputation, and quality of life in patients with DM, it is important to acknowledge certain limitations. The cross-sectional design of this study limits the ability to establish causal relationships between variables. Longitudinal studies would be beneficial in determining temporal relationships and understanding the long-term effects of these complications.

### Recommendations

Healthcare providers should prioritize early detection and screening of peripheral neuropathy in patients with Diabetic mellitus. A multidisciplinary approach involving healthcare professionals from various specialities, such as endocrinology, podiatry, and physical therapy, is recommended, along with long-term follow-up. Collaboration among these specialists can provide comprehensive care, including education, foot care guidance, wound management, and rehabilitation services for individuals with peripheral neuropathy or amputation. Recognizing the impact of peripheral neuropathy and amputation on the quality of life, healthcare providers should offer psychological support and rehabilitation services to affected patients. Access to counselling, prosthetics and mobility aids can help individuals cope with the physical, emotional, and social challenges associated with these complications.

### Limitations

The present study had a few limitations which need to be mentioned here. The study's sample was drawn from patients attending a specific healthcare facility, which may introduce sampling bias and limit the generalizability of the findings to a broader population. Patients seeking care at these facilities may have different characteristics or access to healthcare compared to the general population. Individuals with concurrent chronic illnesses like heart disease, cancer, or renal disorders, with a history of trauma, severe psychiatric conditions, or addiction were excluded due to some ethical limitation. This prevented the analysis from having external validity. Many patients have multiple comorbidities, and it is difficult for neuropathy to be the only complication.

Only MNSI was used, and it could not be compared with nerve conduction study, which is a gold standard for diagnosing patients with peripheral neuropathy. Due to ethical considerations, the interpersonal relationship component of Asian DQOL was not included in this study. The study relied on self-reported data, which was subjected to recall bias. Patients may have inaccurately reported their medical history, symptoms, or quality of life. Additionally, subjective assessments of QoL may be influenced by individual perceptions and experiences.

## Conclusions

Peripheral neuropathy and amputation are typical complications of DM with poor quality of life of affected patients. Our research study aimed to explore the prevalence of peripheral neuropathy, amputation, and the status of the quality of life in patients with DM. To investigate the prevalence of these complications and quality of life, a cross-sectional observational study was conducted using the Michigan Neuropathy Screening Instrument, Asian Diabetic Quality of Life Questionnaire and in order to determine the level of amputation, we incorporated visual aid in the form of a picture. The results indicated a high prevalence of peripheral neuropathy and amputation in the studied population, with a poor quality of life. These findings highlight the importance of early detection and preventive measures and comprehensive management strategies of peripheral neuropathy and amputation in patients with DM to improve their quality of life.

## Data Availability

The datasets used and/or analyzed during the current study are available from the corresponding author upon reasonable request.
